# Integrated Knowledge Translation for Social Innovations: Case Study on Knowledge Translation Innovation Incubator

**DOI:** 10.2196/77581

**Published:** 2026-01-14

**Authors:** Sakiko Yamaguchi, Alix Zerbo, Connie Putterman, Kristine Russell, Roberta Cardoso, Zachary Boychuck, Keiko Shikako

**Affiliations:** 1 School of Physical and Occupational Therapy, Faculty of Medicine and Health Sciences, McGill University, 3630 prom Sir-William-Osler, Montreal, QC, H3G 1Y5, Canada, 1 514-398-3403; 2 Research Institute of the McGill University Health Centre, CHILD-BRIGHT Network, Montreal, QC, Canada; 3 School of Rehabilitation Science, CanChild, McMaster University, Hamilton, ON, Canada; 4 Centre for Addiction and Mental Health, Toronto, ON, Canada; 5 Department of Medicine, McMaster University, Sepsis Canada, Hamilton, ON, Canada

**Keywords:** integrated knowledge translation, innovation, patient-oriented research, disability, case study, research engagement

## Abstract

**Background:**

The Knowledge Translation Innovation Incubator (KTII) initiative, launched by the Knowledge Translation program of the CHILD-BRIGHT Strategy for Patient-Oriented Research Network, provided funding support for researchers and partners to experiment with various approaches and strategies to support the development of innovative knowledge translation (KT) research in the context of neurodevelopmental disabilities.

**Objective:**

We aimed to describe the process and contexts of innovation development in integrated knowledge translation (iKT) practices in patient-oriented research.

**Methods:**

We applied an iKT practice to conduct the collective case study of 7 KTII-funded projects. We interviewed 10 researchers, 4 research trainees, 2 clinicians, 2 parentpartners, 2 patient-partners (1 adult and 1 youth), 1 community partner, 1 KT specialist, 1 designer, and 1 research program manager at the middle and the end of the project period. We conducted qualitative content analysis using the Consolidated Framework for Implementation Research to identify and assess patterns of determinants of (1) drivers of innovation, (2) facilitators and barriers to innovation development, and (3) enablers for sustainability of KT products.

**Results:**

Innovative KT was majorly driven by the identified know-do gap to meet the needs of people with lived experience. Outer setting constructs, such as funding and partnerships and connections, were not only drivers but also facilitators to innovation development. iKT practices presented in this case study were fostered by researchers’ approach to participatory design, involving iterations of listening to emerging ideas and feedback of patient-partners and other partners, and researchers’ continuous reflections on their roles in knowledge creation. Despite the challenges in building consensus and the limited time of the fluid process, researchers’ strong passion for engagement and value placed on lived experience led to flexible engagement and open communication to create KT products. Intangible outcomes included further relationships at individual and organizational levels, capacity building of young people, and a collective voice to influence communities. Sustainment of the KT products requires not only accessibility and adaptability of the product itself but also mechanisms at inner settings, such as training, continued interest of patient-partners and the community, and institutional partnerships to support the further uptake of the product.

**Conclusions:**

This study illustrates the critical roles of researchers in addressing power dynamics and making the research partners’ tacit knowledge visible for successful innovative KT. The research landscape should also change in terms of funding and timeline in order to foster researchers’ mental models in designing thinking and actions on collaborative research engagement.

## Introduction

Research on concepts, theories, and frameworks for knowledge translation (KT) and implementation has rapidly evolved in the past two decades [1]. The Canadian Institutes of Health Research (CIHR) defines KT as “a dynamic and iterative process that includes the synthesis, dissemination, exchange, and ethically sound application of knowledge to improve health, provide more effective health services and products, and strengthen the healthcare system” [2]. One element of KT science focuses on identifying, testing, and developing the best methods to meaningfully engage partners in research and to convey the findings and outcomes of scientific research to those that are interested in or affected by the research. It aims to implement findings and effective evidence-based interventions into health care, policy, and other areas of practice or clinical settings. In Canada, KT is strongly encouraged in the research process since the adoption of the Knowledge to Action Framework in 2006 [3], mainstreamed by the CIHR [4].

The CIHR’s Strategy for Patient-Oriented Research (SPOR) initiative was created to emphasize the engagement with diverse partners in KT and the integration of “patients” as partners in the research process. To this purpose, “integrated knowledge translation” (iKT) has been put forth as a useful model for collaborative research. It is expected that engagement with patient-partners can improve study development and increase uptake of evidence. Despite the recent development in strategies to engage a variety of partners in the research process, challenges still remain: a misfit between the type of problem and the approach taken to address it and a lack of validated methods for research partner engagement in terms of how to measure outcomes of engagement and how to quantify and qualify what meaningful engagement is and what the best methods to conduct studies using this approach are [5,6]. The development of new strategies that address these challenges and evolve with the field of iKT is timely.

The CHILD-BRIGHT Network is a pan-Canadian network that aims to improve life outcomes for children with brain-based developmental disabilities and their families. This network was funded by the Canadian Institutes of Health Research SPOR program, and therefore patient-partners (youth with disabilities and parents or caregivers of children with disabilities) are actively engaged as partners in all research projects and the activities of the network as a whole. The KT program of the network launched the KT Innovation Incubator initiative with the purpose of conceptualizing a vision for iKT, providing funding support for researchers and partners to experiment with various approaches and strategies to propose innovation and support the development of innovative KT research in the context of neurodevelopmental disabilities.

The current research challenge contends that many research engagement approaches are poorly specified and unvalidated [6]. In addition, children and youth with disabilities and their family members are not fully involved in the implementation of health research [7,8]. In this context, it would be beneficial to consider innovations in the process of conducting iKT practices. Innovation is here defined as a product, action, service, or relationship that has the potential to enhance health outcomes [9]. Innovative KT involves multifaceted innovativeness in developing and implementing tools that help the wide dissemination and uptake of new knowledge, engaging with diverse research partners. One example is the translation of evidence-based recommendations in clinical guidelines into educational tools and accessible resources to different target audiences by engaging with key opinion leaders, as well as the creation of a training program [10]. Seven Knowledge Translation Innovation Incubator (KTII) awarded teams had their own visions, approaches, strategies, and relationships for research engagement with diverse partners to bridge the gap between knowledge and practice in a particular context. In this context, this study aimed to describe the process and contexts of innovation development in iKT practices in patient-oriented research.

## Methods

### Research on Research

This study is best understood as research-on-research: a collective case study examining the processes and contexts of innovation within iKT practice happening in the context of 7 KTII projects [11]. We applied a case study, which is “an empirical enquiry that investigates a contemporary phenomenon in depth and within its real-life context, especially when the boundaries between phenomenon and context are not clearly evident” [12]. We describe both the phenomena and the context to gain an in-depth understanding of how innovation happens in patient-oriented research, specifically in the area of neurodevelopmental disability. A collective case study can help us understand the differences and the similarities between the cases (ie, projects) and generate a broader understanding of a particular topic [11-13]. Our constructivist approach aimed to capture the perspectives of different participants and focus on how their different perspectives and meanings illuminate the context and process of innovation development in iKT practices [13]. The comparison between different partners in one case (eg, researcher vs nonresearcher partners) as well as between different cases (ie, projects) was made when mapping the codes on the Consolidated Framework for Implementation Research (CFIR) framework and reviewing the particular contextual information. While qualitative content analysis was used to support thematic synthesis, the primary aim was not theory development or phenomenological inquiry but structured reflection on the research process.

### KT Innovation Incubator Initiative

The KT program launched the KTII initiative with the purpose of conceptualizing a vision for iKT, providing funding support for researchers and partners to experiment with various approaches and strategies to propose innovation and support the development of innovative KT research in the context of neurodevelopmental disabilities. The objective of this initiative was to study how innovation involving “the process of making changes to something established by introducing something new” [14] can be adopted into KT strategies in the context of a patient-oriented research network.

Seven Canadian KT projects were selected to receive funding (CAD $12,000, approximately US $9300 at the conversion rate of US $1 = CAD $1.29 in 2018) from this KTII initiative from 2018 to 2021 in order to promote and facilitate innovative KT products in childhood disability (Table 1). In 2018, the inaugural team, the Child-Sized KT project, proposed to co-design an online family portal that uses child and family partner stories about the value of research engagement. In 2019, the Making Sense of Connectedness project was awarded to work with neurodiverse youth to co-develop initiatives to promote sensory-friendly spaces in Montreal through a web-based hub. The Ready 2 Work team proposed to create an online platform to help young people with autism spectrum disorder successfully enter the workforce. In 2020, the WeeWheel project team aimed to develop and adapt the Wheelchair Skills Training Program educational resources for children through the creation of a training workbook, instructional posters, and a storybook. Another awardee, the Perspectives of Mental Health project, proposed to develop strategies and materials that could facilitate dialogues between youth and health care providers. In 2021, the Let’s Go to the Library! team focused on the voices of young people to design and develop storybooks on different sexuality topics for preteens. Lastly, the CommuniKIDS team proposed to develop a freely accessible bilingual trial results communication tool in collaboration with youth and families impacted by different forms of child disability.

**Table 1.** Overview of the 7 KTII^a^ projects.

Project title	Innovation incubation goal	PWLE^b^	Family or caregivers	Others	KT^c^ approach	Methods	KT products
Child-Sized KT	Develop an interactive online platform for children and families to learn about health research	✓ (children)	✓	Health care providers and writers	Knowledge to Action Framework model	Qualitative interviews and meetings	Family stories and online family portal
WeeWheel	Develop and adapt Wheelchair Skills Training Program education resources for children to address the evidence-practice gap	✓ (children)		Health care providers, decision-makers, and knowledge users	Knowledge to Action Framework model	Focus groups and interviews	A storybook, instructional posters, and a training workbook
Ready 2 Work	Develop and pilot an online vocational/employment readiness platform for people with autism spectrum disorders, families, and vocational program professionals	✓	✓	Advocates and professionals from vocational and employment organizations	Need to Knowledge Model and iKT practice	Focus group, testing, feedback, and piloting	Websites
Making Sense of Connectedness	Give neurodiverse children and youth and their families an opportunity to build an online hub of sensory environments in Montreal to engage the public about the impact of these sensory spaces	✓ (youth)	✓	Community partners (decision-makers from research institutes), students, and designers	iKT practice	Meetings	Pamphlets, videos, bags, and T-shirts
Perspectives of Mental Health	Create digital stories of youth with neurodevelopmental disabilities that can facilitate more dialogue between youth and health care providers in mental health discussions	✓ (youth)	✓	Community partners	Co-KT Framework	Workshops	9 digital stories
Let’s Go to the Library	Create a free book to support nonjudgmental conversations with preteens with disabilities on sexuality and disability	✓ (youth)	✓	Health care providers, educators, graphic designers, multimedia consultants, website developers, professional writers, and actors	iKT practice	Online meetings and the use of information-sharing platforms	Books (downloadable PDF or narrated version)
CommuniKIDS	Develop a freely accessible trial results template in collaboration with youth and family advisors	✓ (youth)	✓	Health care providers or trialists, research ethics board (REB) committee members, and graphic designers	iKT practice	Virtual workshops	Trial results template, tip sheet for template users, and websites

a KTII: Knowledge Translation Innovation Incubator.

b PWLE: people with lived experience.

c KT: knowledge translation.

### Use of Integrated Knowledge Translation in Our Case Study

We also used iKT, a model of collaborative research, to conduct the collective case study of 7 KTII-funded project teams [15]. The KTII funding applications were reviewed by the KT Program review panel, which included a number of researchers, research trainees, and nonresearchers (parents of children with disabilities, youth with disabilities, clinicians, and community partners). Each project was assigned to a dyad of peer reviewers constituted by 1 researcher and 1 nonresearcher, according to the research topic proposed (eg, KT projects directed at families were reviewed by a parent or researcher dyad). All members of the panel participated in the development of the application assessment forms and received equal training to rate applications. While the evaluation grid was used to standardize the rating of applications, each dyad had discussions to clarify their viewpoints and rationale for the rating results to provide the shared review results based on both the researcher and patient or parent-partner perspectives.

Our case study team—consisting of researchers, a project coordinator, parent-partners, and trainees—shared different research tasks throughout the case study series, including cochairing the review panel and addressing questions of panel members. Our parent-partner, who was part of the review panel, contributed to conceptualizing, designing, analyzing, and writing the case study as an integral part of the research team. While the researcher, who co-led the KT program with the parent-partner, guided the data collection and analysis process, both complimented each other’s expertise—the researcher’s expertise on the methodology and the patient-partner’s expertise based on lived experience, along with her curiosity about the topic—and the collegiality enabled shared decision-making during the regular coleads meetings.

### Ethical Considerations

Institutional ethical approval was provided by the Institutional Review Board at McGill University Health Centre-Research Institute (2019-4745). Written informed consent by participants was obtained prior to interviews. Participants did not receive compensation. The persons with lived and living experience who are coauthors were compensated following the CHILD-BRIGHT patient-partner compensation guidelines [16].

### Participants and Data Collection

Participants were members of the KTII-funded projects’ teams. The funding criteria included the inclusion of at least one nonresearcher as coprincipal investigator (including financial compensation for this person and other nonresearcher partners in the study budget description), the submission of a midterm and end-of-grant report that focused on reporting on the KT innovation and iKT methods, and the applicant’s acceptance to participate in the KTII case study.

The studies’ principal investigators and other partners who were members of the research team (not study subjects or participants) participated in two semistructured interviews. The interview guide was developed in partnership with the KT committee members for general input and in detail with the parent-partner, trainee, and researchers who accepted to participate in the specific project subcommittee. Interviews were conducted by a project coordinator at 2 points: midproject and end of the project. The interview at the midproject point focused on the definition of innovation, drivers of innovation, facilitators, barriers, and challenges of innovation development, innovation development process, and engagement with partners. The interview at the end of the project focused on the innovation development process, tangible and intangible outcomes, and sustainability of the developed KT innovation product (Multimedia Appendix 1). The interview recordings were verbatim transcribed for coding.

### Data Analysis

Each KTII project is considered as a case in our analysis. We conducted qualitative content analysis [17,18]. First, a list of codes was cocreated based on the interview questions (eg, driver of innovation, engagement with partners, and enabler for sustainability) with the guidance of the senior researcher. After training on qualitative analysis by the research associate involved in the project with guidance from the senior researcher, parent-partners were paired up with a research trainee for analysis. They met on a regular basis, first with the entire research subcommittee, then with their dyads (parent partner-trainee). During the first meeting done via Zoom, all participants opened one Microsoft Word file (online) while the researcher shared her screen. One person volunteered to read the transcript, and the researcher led the prompts toward deductive coding. For example, she would prompt: “What do you think this is about? Does it speak to any of the items we already have here such as innovation, engagement, sustainability, or is there something else that this participant is communicating? If so, what is it?” The initial codes were done in this fashion, using color codes and comments on the online Microsoft Word document. In parallel, a living document (online shared) of code definitions was created, where written comments prompted discussion for clarification and establishment of the common understanding (referred henceforth as “Journal”). This was done for a series of meetings until the first interview transcript was entirely coded, with breaks to clarification and for any process or content questions from all involved. Then the dyads met to code the same interview transcript and met with the entire group once a month to review what they had coded, including notes, questions, and reflections. While reviewing the results of their partners’ coding and discussing the findings, new codes were added, and the creation date and rationale were added to the journal.

After the iterative process of both deductive and inductive coding, a research trainee reviewed all coded texts and consolidated coding results in NVivo 12 (Lumivero). The preliminary findings were shared with the team members to receive feedback. As the coding process continued, the trainee iteratively reviewed and organized the code list by referring to the updated CFIR [19,20]. The initial data analysis plan did not consider the use of an implementation framework. However, we adopted CFIR during the data analysis as we needed a standardized structure for building on findings across multiple cases, while comprehensively distinguishing a wide spectrum of contextual determinants ranging from external context to individual characteristics [21]. CFIR provides a guiding framework to identify and assess a range of contextual factors of innovation development and implementation in 5 major domains: intervention characteristics, outer setting, inner setting, characteristics of the individuals involved, and the process of implementation. Determinant frameworks applied in CFIR helped us identify and assess patterns of determinants of (1) drivers of innovation, (2) facilitators and barriers to innovation development, and (3) enablers for sustainability of KT products across the intervention development process among different cases [20].

Engagement of parent-partners as well as research trainees shifted during the entire project period due to a shift in roles, personal conditions, and commitments. Although scheduling a meeting specific to the case study became difficult due to everyone’s limited availability, we used regular meetings for the KT program coleads or committee members and email communication within the case study team to report on the progress of the interviews and to discuss the preliminary results of the analysis to ask specific questions and establish confirmability. In addition to the interview transcripts, midterm and final reports submitted by each KTII project were reviewed for data triangulation to gain a more comprehensive understanding of the project context when interpreting findings from interviews [22]. The midterm reports included information about achievements, engagement strategies, innovative KT approaches, and challenges faced by the project team. The final reports included future recommendations. The information validated what had been shared during the interviews, while adding other contextual information (eg, impact of COVID-19 and organizational change) which was not necessarily mentioned during the interviews. The research trainee who reviewed the reports took notes on new information about the process and context of the project. We used strategies to enhance analytic credibility—such as coding dyads, peer debriefing, and triangulation with project reports—but did not apply a full trustworthiness framework, as our aim was not to generate generalizable qualitative findings but to support learning about applied iKT practices.

### Reflexivity

We position our research within social constructivist paradigms, and our stances on reflexivity deeply reflect this paradigm. Social constructivism posits that knowledge is created and applied through individuals’ active interactions and learning in a particular social context [23]. SPOR’s endorsement of the active partnership of research partners, including parent or patient-partners, researchers, health professionals, and decision-makers, shaped our attitude toward the way we as a team created new knowledge based on the shared value of collaboration and colearning. While team members’ educational background and research experience varied, the spirit of colearning and the value of positioning parent-partners as equal research partners created each member’s openness to different perspectives and points of view. The senior researcher learned about a different way of conducting qualitative analysis by partnering with a parent-partner in all steps of the data creation and analysis. This prompted reflections about qualitative methods and true partnered research, which had previously been done mainly on KT processes (eg, dissemination and feedback on outputs), not systematically through the creation of questions, analysis, and manuscript production. The parent-partner, who was the colead of the KT program, appreciated the expert knowledge from a senior researcher who guided the qualitative data analysis. The process gave the parent-partner confidence to contribute. Participating in the KTII case study allowed the research associate to bridge methodological rigor with meaningful partner engagement, ensuring that partners felt confident and supported in the qualitative analysis. The research associate role fostered richer, more nuanced interpretations and strengthened the integration of diverse perspectives in the final results. The project coordinator valued the collaborative nature of the iKT process, which created an adaptive learning environment where research team members not only learned about the different aspects of the research study (eg, qualitative analysis) but also appreciated how meaningful engagement of partners brings about relevant perspectives and enriches the process. The research trainees also appreciated parent-partners’ critical insights into iKT practices, their strong curiosity, and active engagement through bringing questions during coding and analysis. It was also a learning process to reflect on the role of researchers and rethink what makes KT innovative beyond the existing common research practices.

## Results

### Synthesis

Participants included 10 researchers, 4 research trainees, 2 clinicians, 2 parentpartners, 2 patient-partners (1 adult and 1 youth), 1 community partner, 1 KT specialist, 1 designer, and 1 research program manager who were members of the KTII-funded projects’ research teams.

Many participants described outcomes, as well as the approach and process of engagement with research partners in their KT project, as innovative. Innovative KT was majorly driven by the identified know-do gap to meet the needs of people with lived experience. Outer setting constructs, such as funding and partnerships and connections, were not only drivers but also facilitators to innovation development. iKT practices presented in this case study were characterized by researchers’ listening to ideas of patient-partners and other various partners with specific expertise and their continuous reflections on their role in knowledge creation. Despite the challenges in building consensus and limited time, researchers’ strong passion for engagement and value placed on lived experience allowed flexibility of engagement and open communication to create KT products. Intangible outcomes included further relationships at individual and organizational levels, capacity building of young people, and a collective voice to influence communities. Sustainment of the KT products requires not only accessibility and adaptability of the product itself but also mechanisms at inner settings, such as training, continued interest of patient-partners and the community, and institutional partnerships to support the further uptake of the product.

### Drivers of Innovation

Interview participants commonly conceptualized innovations as creativity in thinking and actions under a vision for creating something new for improvement and problem-solving by thinking outside of the box and pushing boundaries. A critical driving factor for innovation development was a construct of the CFIR Inner Setting domain, tension for change, or the degree to which research partners perceive the current situation as intolerable or needing change (Multimedia Appendix 2). Multiple researchers reported that they had identified the evidence-practice gaps to adapt programs and services that are informed by people with lived experience.

In one case, the identified gap was a lack of knowledge uptake since the “wheelchair skills training program isn’t adapted to the pediatric client and the clientele or the pediatric population” [Clinician, Project 4]. Similarly, a researcher in another case (Project 7) stated, “it seemed surprising that nothing like this (communication tool) was available to trialists who wanted to share trial results back to families...and the kids.”

The identified unmet needs driving innovation in 4 cases can be described through a human rights lens or broader issues of injustice toward youth with disabilities (Projects 2, 3, 6, and 7). A project lead researcher (Project 6) stated that “it is a fundamental human right to be able to explore [your] sexuality and be a sexual person in whatever way that looks like for [you]” by referring to young people with disabilities who “don’t have those opportunities to express their sexuality, to figure out their identity.” A parent co-lead in Project 2 also stated, “I think what brought us into here...there are voice to be heard,” by quoting her son, who described the sensory environment where autistic people do not feel welcomed and people’s misunderstanding or ignorance as unfair and injustices. Similarly, a researcher in Project 7 explained why tailoring trial results communication tool to youth was needed because youth themselves “have that autonomy and the right to get the results back from their own trials as well.”

Among the CFIR outer setting factors, funding and partnership and connections were common drivers for innovations. In Project 1, a researcher reported that a series of conversations among different research groups who already had good relationships with each other organically led to a partnership development to create a digital technology innovation. At the inner setting level, institutional strategy to adapt the Wheelchair Skills Program as a relative priority to the pediatric population was an additional innovation driver (Project 4).

In the individual domains, the project lead’s motivation was an often-cited driver of innovation. Researchers in all cases expressed their motivations, passion, and interest in knowledge cocreation with patient-partners during the interview. They also shared their strong belief that lived experience is a valuable source of knowledge that provides a potential solution to the identified complex problem:

I feel like these individuals have some really unique strengths that employers could be utilizing, but we’re having a hard time seeing past that. So, trying to find a platform that not only builds on their current skillset so that they can be seen, but also a platform that may possibly reach employers at some point, be able to see the abilities of this population, and the benefits that they can actually bring to their businesses.

[Researcher, Project 3]

### Process of Innovation Development

#### Teaming, Assessing Needs and Context, and Planning

Most teams applying to this competition had a previously established relationship through ongoing clinical and research activities (eg, research meetings, conferences, and public events). These connections gradually expanded to include other partners, such as family partners, advocates, and designers, to build transdisciplinary teams and pull a project application together. By applying the iKT practice, research partners were involved from the beginning of the project.

At the inner setting, while the needs, priorities, and preferences of patient-partners were broadly identified at the beginning of the project, objectives were not necessarily clear. A researcher (Project 1) stated, “We falsely assumed we knew exactly what we were trying to do, despite having vagueness to what we're trying to do.” The objectives for the project evolved to gradually address unmet needs through a concomitant process of reviewing the existing research evidence and listening to the voices of research partners.

We were listening to our groups. So even if we came up with certain ideas of what we wanted to present, this is like our participatory group here that, you know, our own stakeholders are coming in and saying what they think is important to them. And even like, be it outside consults or our team. And then that helped guide us to where we were going.

[Colead researcher, Project 2]

In the process, many researchers reflected on a shift in thinking of who obtains the most valuable knowledge.

The way I was trained in research was that “We're the experts. We go to them, they tell us how to do it,” but I've found the opposite is true because if we start with them and say, “Okay, these are the things we're interested in. This is what the problem is from a research angle. How do we go about this?”

[Lead researcher, Project 5]

Similarly, a designer and a researcher in Project 2 stated, “the innovation is to flip how we think about expertise, that [young people] are ahead of the game, that they already know these things that would benefit lots of other aspects and people in society.”

#### Tailoring

Once objectives became clear, projects adopted a series of different strategies, such as focus groups, interviews, and regular meetings with project partners, to design innovative products and strategies based on the initial wish list (ie, unmet needs and desires presented by project partners) and available evidence-based resources. After the brainstorming phase, researchers used different strategies to create something useful by integrating what was shared and considering feasibility. In all cases, teams designed a prototype of a KT product or a draft of a KT plan and continued refinement through iterative consultations, member checks, and integrating feedback.

What really helped was...just listening to everybody’s opinions and trying to understand so that...what that does, it allows all the members of the project to add on one another or ask more questions, and so when you ask more questions, it makes the process more exhaustive. So like we truly understand everybody, as opposed to just that small group in the middle that thinks they know what they're talking about, but might not actually understand all of it.

[Youth, Project 5]

Participants expressed the complexity of tailoring the initial design of a KT product to adapt to the needs of patient-partners as a nonlinear process involved a great degree of uncertainty. The capacity to deal with uncertainty and adapt to change was integral inner setting characteristics of many projects.

Never in the iKT process do you see one linear phase of getting to, you know, here’s your research question, here’s the materials, the tools, and then they're up there the everyone starts using. That’s not the way it goes. It’s always this circle of, okay, here’s what we have, we evaluate, here’s what needs to be refined, we bring that back, and it’s always that process of evaluation and follow-up and refining.

[Researcher, Project 4]

During the adaptation of KT products to the partners’ needs, research teams showed the changes made due to the feedback received from research partners. A patient-engagement leader (Project 7) shared:

That was very well received. I mean, people wanna see that. They don't wanna give up on their time to not have an impact. So, for our youth and family stakeholders, I would say that through a combination of evaluating, you know, them, asking them, but also us making sure that we're accountable to them all the time, I think that’s how we know the contribution is making an impact.

[Patient engagement leader, Project 7]

At the same time, outer setting characteristics such as funding and project management posed challenges. Reflecting on the fluidity of KT innovation with partners, a researcher of Project 3 found it a challenge when researchers had to make sure they respected the voices of their research partners while also meeting the expectations of the granting agency or partnership.

In addition, while multiple ideas in the development of projects were highly appreciated, building a consensus with a heterogeneous team was a challenge, as stated in two cases. Key challenges highlighted are related to creating a harmonious balance: (1) between research evidence and innovative elements underpinned by lived experience (Project 6) and (2) between individual preferences and an idea agreed upon by the majority of the team (Project 1). At the same time, a researcher in Project 1 reflected,

I think getting consensus in what we're trying to build, what exactly we're trying to build was [...] probably the biggest challenge. Then so to this question is what’s the biggest successes that [...]. Once we got to that point, things felt very well, which is how it typically [goes] but not always. Sometimes once you start to get along somewhere [during] the iteration, people [start] saying, “Let’s do this, let’s do this,” this sometimes can move [forward] whereas in this one in particular, we got to a point where we drive a process to get to that consensus and now it’s around execution. We had good stakeholder, good feedback and people are engaged. Some of the patient-partners in particular were very helpful.

Even though uncertainty characterized the experienced process of innovation development, it was also considered as an inevitable path leading to discovery, contributing to the adaptation of the intervention being proposed.

You go down a road and you don't know what you're gonna find on that road. So, it was kind of like, “Let’s just do this, and let’s just see what the result is.” So I think that part was really exciting, too.

[Patient engagement leader, Project 7]

Even though things did not necessarily go as initially planned, researchers in the case study commonly highlighted that lived experience guided them during the iterative feedback process.

...youth had come together and...and kind of brought in their...their experience, and what was the best way for them to relay that information that their lived experienced, you know, to the...turn it into a tool that could be useful to others.

[Researcher, Project 2]

Despite the time-consuming nature of the process, it was also a valuable learning experience for many researchers. A researcher in Project 1 stated, “everyone has stuff to learn. We have things to learn about how to communicate better with our family partners.” In Project 3, a researcher explained that a multiple-stage approach was adopted so that people with specific expert knowledge can lead the stage. For instance, “computer tech person will be taking the lead and we [researchers] will be learning from him. So, I think that’s really kind of helpful” [Researcher, Project 3].

#### Engaging

Participants stated that innovation was not only reflected in the KT products created but also in their participatory design process. In many projects, flexibility was a key for active participation of research partners. In three cases (Projects 2, 3, and 5), multiple modes of communication were available so that participants could express their ideas and emotions in a way they would like to. Speaking a lay language was also necessary for researchers’ engagement with patient partners so that everyone on the team remained on the same page. In all cases, researchers also made sure that voices of partners were heard throughout the entire engagement process.

I think that [a parentpartner] said she felt included. We went back and forth in terms of trying to make a decision about something and making sure everyone had input, but she would say, “Well, I defer to you on that because you have the background and quality at research,” but then we’d say, “Well, as a parent, is this going to resonate with you? Or, what do you think is more important? How should we group these things?”

[Researcher, Project 1]

Particularly in cases involving children and young people, several approaches were taken to address inherent adult-youth power differentials. For instance, in Project 7, young people attended meetings led by another youth facilitator, separate from adult research partners.

For the youth meetings, we just have the one person, she’s a young woman, you know, she’s got a rare disease herself and, you know, and she facilitates those meetings and the rest of us turn our cameras off, and we're just in the background. We don't intervene at all. So, it’s a different kind of approach, too. And that’s just a decision that we made.

[Patient engagement leader]

In another project, when young people and their parents attended meetings together, a youth interviewee shared that “it’s more like the parents are backing up what the youth say as opposed to the parents say it for the youth and then the youth just go on with it” [Project 5]. In Project 4, a researcher reported that a video created with a young patient-partner helped reach out to other young participants for recruitment.

Furthermore, reciprocity in research engagement in the form of adequate compensation such as honorarium, opportunities for skills development, and friendship building was also highlighted in 4 cases. In one case (Project 4), a researcher reported that financial compensation encouraged children to participate, while making them feel that they were given an important responsibility based on their knowledge and expertise in wheelchairs. A researcher in Project 2 shared,

Participant 1: Bringing this awareness out into the public, especially the young public, I think it was very good. Very positive effect. They responded wonderfully to it, they were excited I think to see it.

Interviewer: So you have motivated youth.

Participant 1: And vice versa, I now get to design a course around youth mental health for the spring. I won't design it without having a component where those youth have an opportunity to come in and teach the students. So, it equally influences us, maybe that’s the whole...maybe that’s also a part of the innovation, right? It is not a one-way research model. It changes everybody who comes into contact with it in a way, I think.

An often-cited engagement challenge was keeping the team connected. Despite challenges in scheduling meetings, having regular meetings was reported to be helpful in three cases (Projects 3, 6, and 7). In Project 5, where clinicians’ availability was limited, researchers used their routine meetings to present the KT product, which captivated the interest and also made them feel that the tools really met the need that was named by them in previous studies.” In another project (Project 5), a researcher tried to be flexible by telling research partners, “if you can’t make it, come when you can so that everyone who wants to participate can still participate.”

### Facilitators for and Barriers to Innovation Development

At the outer setting level, technology was identified as one of the facilitators for innovation development in three cases as it can enhance connections and engagement (Projects 1, 3, and 5) (Multimedia Appendix 3). In Project 5, a youth partner stated that the digital platform “becomes easier to communicate” and “easier to show other people what we are doing” since “most youth are automatically accustomed to most digital things [...] more reliant upon social media and the kind of network.”

At the inner setting level, multiple relational constructs are reported to have facilitated the innovation development: (1) relational connections, which were built on the previous working relationships in many cases; (2) a culture that values lived experiences and appreciates patient-partners not only as users but also as knowledge creators; (3) transdisciplinary work that fostered collaborations with people from different organizations and disciplines; and (4) open communication that respects diverse viewpoints. The importance of good relations on the team was highlighted, as one researcher (Project 1) described their team as a “group of people who are super flexible, adaptive, [and] rigidity and boundaries weren’t going to work.”

In all cases, multidisciplinary composition of the team brought in a range of expertise and experiences, including (1) researchers, clinicians, community partners, and parent-partners and patient-partners (youth and adults with neurodevelopmental disabilities, children using wheelchairs, and families) and (2) people with specific expertise in fields such as computer programming, data informatics, behavior analysis, and knowledge brokering. Many researchers reflected on the importance of lived experiences and specific expertise and skills, such as communication designing, website designing, and story writing, as important components contributing to the KT innovation.

At the individual level, researchers’ characteristics (perseverance, openness, passion, and being well organized) fostered patient-partner centered culture. In parallel, researchers often discussed that project team members’ strong interest and willingness to make contributions kept the research team motivated to move forward. As one parent-partner (Project 1) stated,

It’s a really strong team and they really have a heart for it. I think it'll just keep growing. Patient-family engagement is just the root of us so much that potential that has to be put in place. I think that’s what they're trying to do very hard.

Three cases (Projects 1, 2, and 7) also highlighted the capability of a knowledge translator and facilitator.

Our ability as a team to translate the youth knowledge was almost simultaneous with [designer] because she was quickly generating. She [...] will come and then she would pick up and then she would help start already the translation...When these youths would see that back again, to see their ideas in this kind of very...this format that’s so official, you know, that it kind of solidified their own and ideas. I think it was really engaging. It was immediate. I think that really helped them to feel like they were part of something that was moving forward as a group.

[Researcher, Project 2]

Many researchers identified timeframe and availability of funds as barriers to innovation development at the outer setting level (Multimedia Appendix 4). A researcher (Project 7) pointed out, “[it is] double edge sword of innovation, right? It’s innovative because it hasn’t been done before, but then that also means that you haven’t got anything to learn from before, so it is taking so much more time and other resources to work through this.” Similarly, as one researcher (Project 2) described it as “reverse order of things,” researchers stressed that the iKT practice cannot be done properly in a conventional research timeline that expects finishing the study and publications within a certain amount of time.

The time I ask [patient-partners] versus the time they give me a response, it could be a few days. It could be a week. Versus if I make that decision on my own, it’s a lot faster, right? So, again, there’s value and merit to that, but the time delay piece, again, in a world so obsessed with being so hyper-productive all the time can lose some of the value of what we're doing.

[Researcher, Project 5]

Therefore, the funder’s flexibility to allow noncost extension was highly appreciated, as a researcher (Project 6) stated, “We have had to extend a couple of times and flexibility has been critical for us to produce this high-quality product.”

Furthermore, 6 project teams were developing innovations during the COVID-19 pandemic (critical incidents at outer settings), which brought unprecedented barriers to innovation development and required creative, flexible thinking and acting on top of the planned innovation process:

COVID happened and COVID just really floored us. I mean, really, really floored us 'cause I think we were making really great strides up until then and then everything changed.

[Parent-partner, Project 2]

The pandemic gravely delayed the ethics approval process and changed the mode of participation from in-person to online. During this unprecedented event, research teams (Project 4) had to be creative to conduct interviews with a child:

Interviewee: What we did to overcome interviewing children online, because of the pandemic, we used a happy face system, um, where if they liked something or thought it was okay or didn't like it, they could do a green happy face, a yellow kind of straight face or a red sad face, or orange sad face. I think it was red.

Interviewer: Yeah, yeah, yeah, yeah angry face or something like that, yeah.

Interviewee: Exactly, and yeah, it worked okay. But the kids wanted to be doing other things. They didn't really wanna be sitting on a screen flashing and sad faces.

Similarly, many interview participants found adaptation due to public health restrictions was a learning opportunity. A researcher (Project 5) described that it was the time to rethink the way they usually conducted research and be creative to make it inclusive. By switching from in-person format to online meetings, improved accessibility for participation was reported in two cases (Projects 3 and 5). It became unnecessary for young people to go to a meeting venue, which in turn opened up possibilities for participation for people in different geographic locations, as well as nonverbal youth participants who were able to engage in discussion by typing their ideas (Project 5).

In addition, limited funding was another barrier at the outer setting. One student (Project 3) described that “we tend to come with these kinds of pie in the sky ideas” when trying to develop something innovative. At the same time, the use of certain technology and hiring people for the development of programs, as well as for administration and coordination, is costly (Projects 1 and 3). In order to manage limited time and budget, some research teams tried to be realistic by selecting areas that everyone had agreed upon (Projects 1 and 2).

### Outcome

In addition to the tangible KT products, many research teams reported additional outcomes had come out of their innovation development process, which they did not expect to see. Several cases (Projects 6 and 7) named new partnerships (outer settings) for further collaboration opportunities.

Given that the organization that I'm representing here, [institution’s name], has now worked with this particular group, I can see us working together on other projects moving into the future too. So while we delivered on the original intended outputs, I think we've kind of seeded things to maybe do other things together as a group.

[Researcher, Project 7]

Such connections were being made outside of research settings in two cases. In Project 2, researchers were excited to see the community members starting to reach out to invite youth groups for consultations. Youth’s friendships were organically fostered in Project 5.

The project teams involving youth research partners highlighted opportunities for capacity building and empowerment (Projects 2, 5, and 7).

Some of the youth have said, you know, like “I showed this to some of my friends who have mental health challenges and have a neurodevelopmental disorder and they never thought that a kid with autism can do this”, right? So again, it’s almost breaking stereotypes for some kids as well that they're not broken or damaged, like they've been told before, but that they have kind of potential and are worthwhile.

[Researcher, Project 5]

In addition, another case stressed the collective voice of youth as the outcome that has the most potential for impact on the community (Project 2).

Researcher: You know, having like your pamphlets and so on. You're not just someone who sounds like, "Oh, I'm advocating for myself or I'm complaining." That’s how people sometimes see you. But coming together as a collective and having it branded and having it, you know, sort of bringing in that credibility, you know, it brings in more gravitas. You have, you know, people’s ear. And so I thought that was quite significant.

### Sustainability of Developed Innovation

The identified enablers of sustainability of the innovation products that each research team developed are multifaceted (Multimedia Appendix 5). In the innovation domains, accessibility and adaptability of the product to different populations were identified as a key enabler for the sustainable implementation of the innovation. Whereas a strategy to make materials available online through their own website or their partner organizations’ website was put in place in many cases, one research project (Project 4) also pointed out the need to print resources in both a print and a digital version to share with families as well as clinicians.

Furthermore, innovation was seen in the ways that teams granted credibility to their KT products. In one case (Project 6), they obtained an ISBN as a strategy to increase the sustainable use of their book. They explained: “it helps [a library at our hospitals] to catalog our book and for us, it helps get the book out, so it is sort of both adding credibility but also helping other people get it out more.” Another crucial enabler was funding to update and maintain the developed product relevant to the users and/or expand the users to different target groups (outer settings).

In the inner setting domains, in Project 4, whose target users include clinicians, researchers were aware of the need for training for implementation. Therefore, continuing education on the innovation (pediatric wheelchair skills training) was a work infrastructure in inner settings that was required to make a longer use of the developed KT innovation.

Some research teams found relational connections with existing and newly developed partnerships with other research teams as an enabler to innovation and help sustain the developed innovation products. For instance, a researcher (Project 7) stated, “even though the project team is formally disbanded, there is a commitment from [the name of an institute] as a partner organization to continue to update the website, and to continue to potentially make changes to the results template if we’re hearing enough feedback from people that that should be done.” Similarly, another project team (Project 6) believed that close relationships with the communications and public engagement department, as well as a very large network of partners, can help disseminate their KT product and guarantee better knowledge uptake and use.

Many project teams also found that continued interest of patient-partners and the community, which were part of the innovation development process, can help sustainable use of the developed product.

I'm looking forward to those benefits that I think will come as we build a community of people who are involved and actively participating on the [web]site because again, I'm the researcher and [...] I see my role as facilitating the process but it’s meant to live as a result of the community who benefits from it.

[Researcher, Project 3]

The team of Project 2 discussed that sustainability is not just the product but also relationships to create changes in the community:

I do and that, you know, usually, we think of sustainability, like as an environmental or the longevity of a product, but it’s essentially grounded in our relationships, right? And if people are empowered...I think you were both saying that P3 and P2 in different ways, like to...that they just know that they can create these changes. I mean, I think that’s what we're trying to give people more than any actual product in a way.

[Researcher]

On the other hand, local attitudes in outer settings can be a potential barrier to the sustainable use of the KT product. The members of Project 3, who developed an online platform that provides resources and tools to people with autism in order to support their employment, noted that “we need to start challenging employers’ perceptions of individuals with autism” by seeing them facing the structural barriers to employment. Furthermore, they also found that maintaining relational connections and networks that are aimed to be created by the developed platform can be a barrier to sustainable use, as people’s needs can shift while the employment situation is always changing.

## Discussion

### Interconnected Contextual Factors

**Figure 1.**
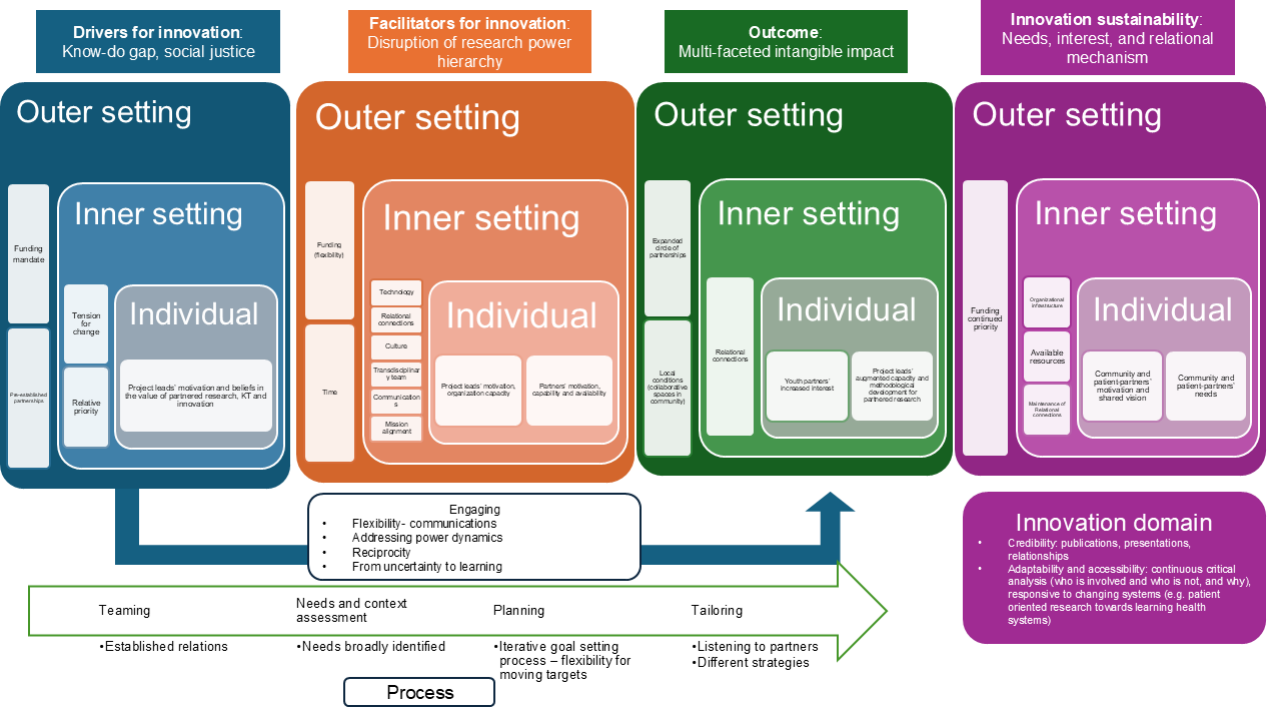
Overall findings with CFIR constructs. CFIR: Consolidated Framework for Implementation Research.

A range of contextual factors in different domains of the CFIR framework (outer settings, inner settings, and individual characteristics) are interconnected to shape the unique process of innovation development in each case (Figure 1).

### Driver for Innovation: Closing the Know-Do Gap

In addition to resources such as funding and existing partnerships, identification of a clear know-do gap was a major driver for innovation development [24,25]. In four cases (Projects 2, 3, 6, and 7), a social justice lens focusing on the human rights of young people with disabilities was a driver for innovation development. Although little attention has been paid to social justice and equity in iKT discourses [24-26], these cases demonstrate that social justice can be a critical starting point for KT efforts to advance health equity.

Another critical driver for innovation development was researchers’ attitude toward knowledge cocreation [27]. While the know-do gap was historically conceptualized as a problem of knowledge transfer (for instance, inadequate efforts to translate academic knowledge into practice), an iKT model considers the know-do gap as a problem of knowledge production [25]. In all cases, interviews reflected researchers’ beliefs and philosophies in research partnership with patient-partners, which were also identified as facilitators for innovation development, and not only knowledge users.

### Participatory Design in the Innovation Development Process

All research teams applied a participatory design approach where “participants are not only research subjects but also contributors to the design of a service of other outcome that will affect them” from the beginning of the project [28]. The collaborative process led to the creation of spaces where different types of knowledge were valued and shared, and solutions to address pressing real-world challenges were collectively created [29-33]. However, the existing hierarchies of value in knowledge systems are constructed against a background of social and institutional relations and cultural context [24,31,34]. Therefore, patient-partners’ voices can be often neglected due to power imbalances or methodological structures for generating “valid” knowledge [35,36]. The issues of power imbalance encountered in the cases of this study were attenuated by a funding and reporting structure that valued and, in a certain way, regulated a collaborative and more equal structure [30].

This study highlighted the critical roles of researchers in making the research partners’ tacit knowledge visible and turning the KT process into “collective making” [30]. The researchers’ openness and listening to diverse views, respectful and accessible communication, and provision of multiple methods of participation facilitated relational connections and the team culture that recognizes people with lived experience as valuable knowledge partners [37]. Researchers also made intentional efforts to address the existing power difference by having a youth or a peer facilitator [35].

For some researchers in this case study, building consensus was not easy due to tensions of leveraging lived experience [38,39]. Nonetheless, they made the cocreation process accountable, transparent, and authentic by showing the changes made based on their input and acknowledging their contributions [30].

The space of “collective making” was gradually built by generating research partners’ interest in the process, as well as having knowledge translators, facilitators, and specialists, such as IT specialists as knowledge brokers.

The traditional knowledge-to-action approach tends to hold linear assumptions that knowledge comes first, and it underlies effective action and practices [40]. By contrast, in this study, uncertainty was an inevitable part of the process yielding innovations, requiring researchers’ openness to changes and funders’ flexibility.

### Outcomes

In addition to the tangible KT products, all cases have reported different types of other intangible outcomes, including expanded research relationships that can be leveraged for knowledge mobilization and further research opportunities, as the knowledge cocreation process became a “relational design” [41]. Considering the transformational aspect of the iKT practice leading to innovation sustainment, we posit that capacity building and empowerment through research engagement and raising awareness through community engagement should be considered as the iKT’s primary goal for effective knowledge uptake and sustainment of knowledge application [37,41].

### Keeping the Innovation Sustainable

While accessibility and availability are commonly identified as the key to sustainable use and implementation of the innovative product, a variety of funding should be available since human and financial resources are necessary to keep the knowledge updated and accessible. The innovation sustainment often requires changes in local conditions and attitude (outer settings) to create a favorable socioeconomic environment to address inequality and injustices that people with lived experience are facing in their daily lives and in the health system. Therefore, including a strategy to bring a positive change in the local conditions and attitudes through community engagement is important during the creation of KT products [42].

### Implications

While several recommendations for forming and maintaining research partnerships are already drawn and presented somewhere else [43], this case study using the CFIR highlights that iKT practices require additional time, effort, and resources for a long-term engagement with research partners [44]. To support the relationship building, iterative participatory design process, and sustainable uptake and use of the product, we recommend flexibility and diversity of funding [5]. We also suggest that funding, reporting, and regulatory structures are put in place to allow for projects to develop in a context of uncertainty, but having the collaborations and partnerships at the center of the requirements.

In parallel, uncritical emphasis on participation without a shift in power dynamics may pose a risk of turning iKT into a new label for tokenistic research relationships [45]. In this case study, researchers were reflective of whose voice is missing, and their characteristics mediated to foster a positive team culture that values lived experiences as expert knowledge. This finding reiterates the importance of a shift in researchers’ mental models, as defined as “particular set of conceptual knowledge, expectations, and causal beliefs,” in KT [46]. While academics are not traditionally trained or rewarded for their interpersonal skills, their design thinking, “a problem-solving approach that emphasizes empathy, collaboration, and iterative prototyping to develop innovative and human-centered solutions,” should be better valued in academia [32,44,47,48].

### Limitations

This study has limitations. Even though a total of 24 participants were interviewed from the KTII-awarded projects, the participation of patient-partners was limited. Researchers often felt that they had asked enough of their partners, and participating in one additional interview could be onerous. This is an important consideration for mandates for partner engagement in research. More first-hand accounts of patient-partners as co-knowledge creators, particularly children and young people, as well as information about group-level demographics of interview participants, might have provided a novel and in-depth understanding of effective engagement approaches and processes for innovation development. Case studies including intersectionalities, such as Indigenous research partnerships, would also be beneficial to learn how a transcultural lens can be applied to decolonize iKT practices and to define what we consider innovation and how we respond to needs in different contexts and populations. In addition, all KTII-awarded teams had established research relationships at the time of grant application. Therefore, even though inclusion and equality underlie participatory design [28], critical examination of structural participation barriers related to diversity, inclusion, and representation was limited. Lastly, the collected data did not necessarily include the long-term impact of innovations after the knowledge dissemination activities were concluded. Future studies should also measure the long-term knowledge uptake and its impact on social and health conditions and the sustainability of research partnerships with diverse teams of partners.

### Conclusions

This case study showed multidimensional aspects of innovative KT in patient-oriented research, particularly (1) a clear know-do gap is an opportunity for innovations, (2) innovation is a process as well as an approach of creating new knowledge from lived experience and other expertise of various research partners, (3) innovation disrupts the traditional knowledge hierarchy and power imbalance in research, (4) innovation requires flexibility in timeframe and funding, (5) a challenge can be an opportunity for another innovation, and (6) innovation can bring not only tangible but also intangible outcomes at individual, organizational, and community levels. For successful innovative KT, the research landscape should also change in terms of funding and timeline in order to foster researchers’ mental models in designing thinking and actions on collaborative research engagement.

## Supplementary material

10.2196/77581 Multimedia Appendix 1 Interview guide questions.

10.2196/77581 Multimedia Appendix 2 Drivers for innovations.

10.2196/77581 Multimedia Appendix 3 Facilitators for innovations.

10.2196/77581 Multimedia Appendix 4 Barriers to and challenges with innovation development.

10.2196/77581 Multimedia Appendix 5 Enablers to sustainable use of the knowledge translation product.

10.2196/77581 Checklist 1 GRIPP2 (Guidance for Reporting Involvement of Patients and the Public, second version) checklist.

## Abbreviations

CFIR: Consolidated Framework for Implementation Research
CIHR: Canadian Institutes of Health Research
iKT: integrated knowledge translation
KT: knowledge translation
KTII: Knowledge Translation Innovation Incubator
SPOR: Strategy for Patient-Oriented Research
