# Supplementary material for: Integrated Knowledge Translation for Social Innovations: Case Study on Knowledge Translation Innovation Incubator
Source: J Particip Med. 2026 Jan 14;18:e77581. doi: 10.2196/77581 (PMC12803437; doi:10.2196/77581)
Supplement: Multimedia Appendix 1 [file jopm-v18-e77581-s001.docx]

**Interview guide questions**

Mid-term point

In your own words, how would you define what is “innovative”?

Why do you think this project is innovative?

Why was the project/innovation necessary?

What are the main outcomes of your innovation?

How you would describe it?

How did you go about developing the project?

More specifically, how did you go about developing the innovative aspects of it?

1. How do stakeholders shape the development of strategies, methods, and application of knowledge? How do they know their contribution is making an impact?

What challenges did you face in the project development process?

How did you address or overcome these challenges?

What facilitated or helped the process of developing this project/innovation?

What would be the best-case scenario to fully develop the project/innovation?

More specifically, what would make it sustainable?

Do you believe your research project development process can be used by others to develop innovative projects in KT?

Why?

How?

End-point

1. Based on what you've done so far and being at the end of your projects, in your own words, how would you define what's innovative?
2. What are the tangible outcomes and intangible outcomes that came out of this project? And are they for whom?
3. Do you think these outcomes actually address the problems/issues that your innovation has aimed to address? If so, in what way?
4. How did you go about developing the innovative aspect of your project?
5. How did interested parties shape the development of strategies, methods, and application of knowledge?
6. What challenges did you face in the development process, and how would you do things differently this time? If you had done things differently, how do you think the outcome would be different?
7. What facilitated the process of developing this project/innovation?
8. What would be the best-case scenario to fully develop the innovation?

More specifically, what would make it sustainable?

1. Do you believe your research project development process can be used by others to develop innovative projects in KT?  If so, how?
