# Supplementary material for: Integrated Knowledge Translation for Social Innovations: Case Study on Knowledge Translation Innovation Incubator
Source: J Particip Med. 2026 Jan 14;18:e77581. doi: 10.2196/77581 (PMC12803437; doi:10.2196/77581)
Supplement: Multimedia Appendix 2 [file jopm-v18-e77581-s002.docx]

Drivers of innovations

| **CFIR Domain** | **CFIR Construct** | **Reported cases**  **(Project No)** | **Reflective quotes** |
| --- | --- | --- | --- |
| Outer settings | Local conditions: Physical proximity to partner organizations | Project 1 | Interviewee: We're a partner on site.  Interviewer: Yes. That's very helpful to be on site.  Interviewee: Yes, and that's the best part it because we're here to do that. I think that's how initially it came through. Organically, we say, let's explore this, we will have an initial discussion. (Project 1, researcher) |
|  | Partnership & connections | Project 1, 4, 7 | I think that was [patient-partner’s] involvement and the openness of [institution’s name] to share or what worked well for them and their methods for what worked well for them (Project 7 Program manager) |
|  | Funding | Project 1, 3, 4, 7 | Honestly, I feel like all we need is funding. The drive, the passion, the expertise is all here...The vision, the patience, like everyone is…it’s ready. It just needs money behind it.  (Project 1, researcher)  it's great that we have the funding for this project, but actually, it was a pretty ambitious project (Project 4, research trainee) |
| Inner setting | Tension for change: Know-do gap | All projects | And when we looked at just sort of what was available in terms of an online platform, there was nothing really specifically available to people with autism. (Project 3, researcher)    This is a gap in the field of pediatric disability, so the lack of resources that are available speaks to the need to try to create a series of different resources (Project 6, researcher) |
|  | Relative priority: Strategic priority | Project 1, 4 | They’re taking place at xx Rehabilitation Centre and two of its affiliated schools, a primary school and a secondary school or high school, I should say. and it’s actually a strategic priority right now within the positioning and mobility department at XX Rehab Center. (Project 4, researcher) |
| Individual | Research lead’s motivation: interest, value, fundamental philosophy about partnerships | All cases | We spend so much time, you know, becoming "experts" in this area, sometimes we forget how our research priorities differ from the priorities of the youth that we're working with (Project 5, researcher) |
