# Supplementary material for: Integrated Knowledge Translation for Social Innovations: Case Study on Knowledge Translation Innovation Incubator
Source: J Particip Med. 2026 Jan 14;18:e77581. doi: 10.2196/77581 (PMC12803437; doi:10.2196/77581)
Supplement: Multimedia Appendix 3 [file jopm-v18-e77581-s003.docx]

Facilitators for innovation

| **CFIR Domain** | **CFIR Construct** | **Reported cases**  **(Project No)** | **Reflective quotes** |
| --- | --- | --- | --- |
| Outer settings | Funding | Project 4, 6 | With the funding for this project we’re literally just at the first four or five skills. So we need additional funding, additional resources, time, et cetera, in order to be able to continue this process (Project 4, researcher) |
|  | Time | Project 4, 6 | we've had to extend a couple of times and that flexibility has been critical for us to produce this high quality product (Project 4, researcher) |
| Inner settings | Structural Characteristics: Technology | Project 1, 3, 4, 5 | the Zoom and, you know, all of the technology that has definitely helped. I think we’re getting so used to using it now (Project 4, researcher) |
|  | Relational connections: Team foundations with previous working experience | Project 1, 2, 3, 7, | Like, because you-you and I have done a project, two-two projects before (Project 2, researcher) |
|  | Communication: Open communication, respect | All projects | Having well-established relationships between organizations and, uh, a collaborative mindset and respect for the various roles that both researchers and professionals in the field are doing respectively and making sure that they continue to work together (Project 3, community partner) |
|  | Culture: Transdisciplinary work facilitation | Project 1, 2, 3, 4, 5, 7 | Taking client and family-centered approach is part of our culture, so, um, I definitely say that was a facilitator in terms of bringing-- helping to bring, um, the people who are passionate about this work on board to help them be part of it (Project 7, KT specialist) |
|  | Relational connections: Good relationships | All projects | we get together every two weeks and we work on this project together and everyone has a voice and everyone is contributing so actively (Project 6, researcher) |
|  | Mission alignment: Common goals | Project 1, 2, 7 | we should really align and whatever animation and visuals and images we have in the story…That's how we got into that to make sure the branding and the images are very similar across the different projects because they're aiming to do the same thing (Project 1, researcher) |
| Individual | Research lead’s motivation: interest, value, fundamental philosophy about collaborative partnerships | All | So I think, passion to make a change in all of the stakeholders and in all of the investigators is-is key. Um, probably, especially because it does take a sig-- significant amount of their own time (Project 3, community partner)  Not just like, "Oh, we'll do a project. This is going to happen." How can you make this live on? How can you really change an environment? How can you really change people's perspectives? And so there's different ways of hearing the lived experience, you know, through photographs, through sounds, through whatever, you know, like there's different- there's different ways that people could express themselves.  And here we were listening to our groups. (Project 2, researcher) |
|  | Ability: Manager’s organization | Project 5, 7 | so the perseverance by our lead investigators really, really helps in that aspect (Project 5, youth) |
|  | Capability: Other experts (Knowledge translator/facilitators/designer/multimedia specialist etc.) | Project 1, 2, 6, 7 | (Designer) will come and then she would pick up and then she would help start already the translation. And then, when these youths would see that back again, to see their ideas in this format that's so official, you know, that it kind of solidified their own and ideas. I think it was really engaging. It was immediate (Project 2, Researcher). |
|  | Capability: students | Project 2, 4 | we have our regular monthly meetings. We have the students who have created these three KT tools (Project 4, researcher). |
|  | Motivation: Partners’ interest and willingness to contribute | All | the clinical patient partner was also able to help us by giving us also the clues on to what is needed, what is interpreted by the pediatric population, what is understood by the pediatric population (Project 4, clinician) |
