# Supplementary material for: Integrated Knowledge Translation for Social Innovations: Case Study on Knowledge Translation Innovation Incubator
Source: J Particip Med. 2026 Jan 14;18:e77581. doi: 10.2196/77581 (PMC12803437; doi:10.2196/77581)
Supplement: Multimedia Appendix 4 [file jopm-v18-e77581-s004.docx]

Barriers to/challenges with innovation development

| **CFIR Domain** | **CFIR Construct** | **Reported cases**  **(Project No)** | **Reflective quotes** |
| --- | --- | --- | --- |
| Outer settings | Funding: Limited funding | Project 1, 2 | We're not going to have a functioning platform because we haven't had the resources to really develop that (Project 1, researcher) |
|  | Time: timeframe/timing | Project 2, 5 | I think our biggest challenge in research is this race against the clock, which is stressful because there's always a deadline and there's always expectations (Project 5, researcher) |
|  | Critical incidents: COVID-19 | All project except Project 1 | then COVID happened and COVID just really floored us. I mean, really really floored us 'cause I think we were making really great strides up until then and then everything changed (Project 2, parent co-lead) |
