# Supplementary material for: Integrated Knowledge Translation for Social Innovations: Case Study on Knowledge Translation Innovation Incubator
Source: J Particip Med. 2026 Jan 14;18:e77581. doi: 10.2196/77581 (PMC12803437; doi:10.2196/77581)
Supplement: Multimedia Appendix 5 [file jopm-v18-e77581-s005.docx]

Enablers to sustainable use of the KT product

| **CFIR Domain** | **CFIR Construct** | **Reported cases**  **(Project No)** | **Reflective quotes** |
| --- | --- | --- | --- |
| Innovation domain | Innovation source: Credibility | Project 6 | And we've been able to get like an ISBN number for it to kind of really raise its credibility…ISBN are numbers that are assigned to books, so it's how the product is cataloged within libraries, and typically they're assigned to books, but they can be assigned to a variety of different products, but this was a step that we wanted to take to help facilitate also like our dissemination strategy of where the book can go (Project 6, KT specialist). |
|  | Innovation adaptability: accessibility | Project 1, 3, 4, 6, 7 | so I think to make it sustainable, we need to print the resources, the tools, have them available in both a print and a PDF a digital version to be able to share with families and with clinicians (Project 4, student)  I think there's an element of sustainability there in terms of these resources are gonna be housed on a website where individuals can have access to, you know, dissemination efforts will be put into place for this whole scope of work (Project 6, researcher) |
| Outer settings | Funding: Maintain and update | Project 1, 3, 4, 7 | What would make it sustainable?  Interviewee: Well, there's the obvious funding. We've had unlimited supply of funds to keep creating these tools, and make new tools, and evaluate them. (Project 4, researcher) |
| Inner settings | Work infrastructure: Continuing clinical education | Project 4 | that's something we've heard a lot in other research, but we're hearing from clinicians and physical education teachers and people who were developing tools and programs to be used, but without training or ongoing access to having somebody respond to questions, it's really hard for them to implement (Project 4, student) |
|  | Work infrastructure: Organizational structure, support, and function | Project 1, 4, 6 | The XXX lab, which is her lab that is doing sexuality and childhood disability is an area of [researcher’s name]'s research, right? So I think is already the foundation for sustainability in terms of addressing the topic (Project 6, KT specialist)  I think the development of this sub-committee is gonna help to make this whole process of implementation of the Wheelchair Skills Program in pediatric settings more sustainable (Project 4, Researcher) |
|  | Funding: compensation | Project 5 | some volunteer work is nice, but at the same time, all these parents won't complain if they get reimbursed or are being really adequately kind of paid for their time. So, to really make this work long term, it really has to be like big pictures level from our end to make sure that you have capacity but to also make sure that those who are involved, they're also being taken care of (Project 5, researcher) |
|  | Relational connections: Existing and built relationships | Project 2, 6, 7 | we think of sustainability, like as an environmental or the longevity of a product, but it's essentially grounded in our relationships, right? And if people are empowered, they just know that they can create these changes. (Project 2, researcher)  So this, I imagine that this will also be on the CommuniKIDS website, but it will also be on the XXX website along with the other templates, as I mentioned that we have created. And it will be part of a larger project that we also, or resource kit that we created (Project 7, researcher). |
| Individual | Motivation: Community people’s/partners’ continued interest | Project 1, 2, 3, 7 | I can foresee some of the people who have been involved in giving us feedback on the website have expressed an interest in continuing to be part of it as it sort of, you know, lives somewhere more permanently which is ideal for me because, I mean, for a lot of what we've learned out of this project is very community-driven (Project 3, researcher) |
|  | Needs: Users’ needs | Project 3, 4 | I think if clinicians and children start to use the materials and show there is a need for it, I think, or a want for it, then I think that can make it more sustainable (Project 3, researcher) |
