# Supplementary material for: Integrated Knowledge Translation for Social Innovations: Case Study on Knowledge Translation Innovation Incubator
Source: J Particip Med. 2026 Jan 14;18:e77581. doi: 10.2196/77581 (PMC12803437; doi:10.2196/77581)
Supplement: Checklist 1 [file jopm-v18-e77581-s006.docx]

GRIPP2 Reporting Checklist-Short Form

| Section and topic | Item |
| --- | --- |
| 1.Aim | Engagement with parent-partners in the KTII/case study was essential to ensure that partner perspectives were integrated to inform the implementation of KTII, analysis and interpretation of findings to better understand the process and context of innovate KT research. |
| 2.Methods | Patient/family-partners were involved as a review panel to evaluate the KTII application forms in dyads with researchers. Case study team had one parent co-lead who conceptualized the KT incubation innovator and who helped coding and analysis process in collaboration with researchers. The initial analytical framework was created together. Another parent-partner joined the coding process. Coding was done in dyads of researchers and non-researchers. The team continued coding and discussion through meetings/e-mail. |
| 3.Results | Preliminary findings were shared during the analysis phase. Parent partner co-lead provided her comments, highlighting some critical aspects of iKT practices that she had identified, while reflecting on her own experiences in collaborating with researchers. |
| 4.Discussion/conclusions | While a research trainee drafted the manuscript, a parent co-lead’s comments reviewed it and helped add clarity and perspectives of research partners on how iKT practices can be improved by recognizing various contextual factors presented through analysis. |
| 5.Reflections/critical perspectives | The existing positive relations between a parent partner co-lead and a researcher co-lead created supportive environment for the case study team to collaborate. Open communication, listening to each other, and giving space to share different ideas facilitated the entire project process. As the data collection period took long, each individual’s role/commitment/availability changed. As a result, it became difficult to maintain consistent engagement. However, using regular KT program meetings and e-mails helped everyone keep informed and updated about the progress. |
